# Pharmacological Actions of Potassium Channel Openers on Voltage-Gated Potassium Channels

**DOI:** 10.3390/ph18101446

**Published:** 2025-09-26

**Authors:** Michael T. McCoy, Bruce Ladenheim, Jean Lud Cadet, Atul P. Daiwile

**Affiliations:** Molecular Neuropsychiatry Research Branch, NIDA Intramural Research Program, Baltimore, MD 21224, USA; mmccoy@intra.nida.nih.gov (M.T.M.); bnlgsp12@gmail.com (B.L.); jcadet@intra.nida.nih.gov (J.L.C.)

**Keywords:** potassium channel opener, flupirtine, diazoxide, voltage-gated potassium channels, gene expression

## Abstract

**Background/Objectives:** Potassium (K^+^) channels are essential transmembrane proteins that regulate ion flow, playing a critical role in regulating action potentials and neuronal transmission. Although K^+^ channel openers (agonists, K^+^ Ag) are widely used in treating neurological and psychiatric disorders, their precise mechanisms of action remain unclear. Our study explored how K^+^ channel openers might influence the expression of voltage-gated K^+^ channels (Kv) in rat brain. **Methods**: Briefly, eight rats per group received intraperitoneal injections of diazoxide (Dia), chlorzoxazone (Chl), or flupirtine (Flu). Two hours post-injection, the prefrontal cortex (PFC), nucleus accumbens (NAc), dorsal striatum (dSTR), dorsal hippocampus (dHIP), and ventral hippocampus (vHIP) were collected for mRNA expression analysis of various Kv. **Results**: Dia administration altered expression of *Kcna6* in the NAc, dSTR, and vHIP, and *Kcnq2* in the PFC, dSTR, and dHIP. The mRNA levels of *Kcna2* and *Kcna3* changed in the NAc, dHIP, and vHIP, while *Kcna6* expression increased in the PFC, dHIP, and vHIP of rats treated with Chl. Injection of Flu resulted in altered expression for *Kcna1* in the NAc, dSTR, and dHIP; *Kcna3* in the PFC, NAc, dHIP, and vHIP; *Kcna6* in the dSTR, dHIP, and vHIP; and *Kcnq2* and *Kcnq3* in the PFC, dHIP, and vHIP. We also found dose-dependent changes. **Conclusions**: To our knowledge, this is the first study to identify the effects of potassium channel openers on gene expression within the mesocorticolimbic and nigrostriatal dopaminergic systems. These findings reveal a novel molecular mechanism underlying the action of these drugs in the brain. Importantly, our results have broader implications for translational neuroscience, particularly in the context of repurposing FDA-approved drugs, such as diazoxide and chlorzoxazone, for the treatment of neurological disorders.

## 1. Introduction

Potassium channel openers (agonists, K^+^ Ag) have long been used for the treatment of various disorders: acute and chronic pain [1]; hypoglycemia, acute hypertension [2]; epilepsy [3,4]; and stroke [5]. Their mechanism of action in the brain is poorly understood. In view of this, we used three different potassium channel openers to see their effects on the expression pattern of voltage-gated potassium channels in the rat brain.

Potassium channels (K^+^ channels) are integral pore-forming transmembrane proteins that selectively control the influx and efflux of important physiological ions, including Na^+^, K^+^, Ca^2+^, and Cl^−^, into cells and intracellular organelles. The K^+^ channels have been divided into subgroups based on their molecular structures and mechanisms of activation [6,7]. These include (1) voltage gated (Kv), (2) calcium activated (KCa), (3) two/tandem-pore domain (K2p) subgroup, and (4) inwardly rectifying (Kir) subgroup. We targeted the largest subgroup of potassium channels consisting of voltage-gated (Kv) channels that include 80 genes in 12 families [8]. This family is modulated by subunit compositions (homo- or hetero-polypeptides), their localization, and membrane potential in the cell [9]. They detect voltage changes in the cell membrane by the voltage sensor domain that is coupled to the pore-gate domain [10]. The majority of Kv family genes (Kv1–Kv12) have been found in the brain [11], where they control cytoplasmic and intraorganellar ion concentrations as well as regulate action potential propagation and neurotransmission (for review, see [12]).

Herein, we tested pharmacological agents, specifically the potassium channel openers flupirtine, diazoxide, and chlorzoxazone, to identify their effect on the expression pattern of Kv channels in different brain regions. The first is flupirtine (Flu); this was used for its analgesic actions and muscle relaxant properties via regulating the opening of voltage-gated potassium channels KCNQ2/3 (Kv7.2/3), but negative interactions caused it to be removed from clinical use [13,14]. Next is diazoxide (Dia), which is used in the treatment of acute hypertension and inhibits insulin secretion [2]. The following inwardly rectifying K^+^ channels are influenced by Dia: KCNJ1/ROMK2 [15]; Kcnj1/Kir1.1; Kcnj3/Kir3.1; Kcnj5/Kir3.4 [16]; Kcnj8/uKATP-1 [17]; and Kcnj11/Kir6.2 [2,18]. Lastly, chlorzoxazone (Chl), a Kcnma1/large-conductance calcium-dependent potassium (BKCa) channel agonist, is used as a muscle relaxant [19] and pain relief medication [20,21].

We intentionally looked at gene expression of five out of twelve Kv subfamily members in the rat brain. First, we studied KCNA/Kv1, *Kcna1*, *Kcna2*, *Kcna3*, *Kcna4*, *Kcna5*, and *Kcna6* because of their involvement in neuronal excitability, epilepsy, and other neurological disorders [8]. Next was KCNB/Kv2, *Kcnb1*, *Kcnb2*, and *Kcnb3*; it was shown that chronic intermittent ethanol use changes *Kcnb1* expression in the prefrontal cortex (PFC) [22]. Later, we studied KCND/Kv4, *Kcnd1*, *Kcnd2*, and *Kcnd3*, since *Kcnd2* has recently been reported to be involved in ethanol abuse [22]. Moreover, KCNG/Kv6, *Kcng1, Kcng2*, *Kcng3*, and *Kcng4* were found to be risk variants for opioid dependance in humans [23]. Finally, KCNQ/Kv7, *Kcnq1*, *Kcnq2*, *Kcnq3*, and *Kcnq4* were earlier described; Flu regulates cell excitability by opening the voltage-gated potassium channels KCNQ2/3 [24,25,26].

We studied the effect of potassium channel openers in the prefrontal cortex, nucleus accumbens (NAc), dorsal striatum (dSTR), dorsal hippocampus (dHIP), and ventral hippocampus (vHIP). The PFC plays an important role in the regulation of self-control, decision making, and ingestive and social behaviors [27,28,29,30,31]. The NAc is thought to participate in the regulation of motivated behavior [32]. The dSTR is involved in habit formation [33]. The HIP is an important structure for learning and memory formation [34,35]. Finally, this article examines gene expression patterns of voltage-gated potassium channels and proposes the possible mechanisms of action for the three potassium channel openers flupirtine, diazoxide, and chlorzoxazone.

## 2. Results

First, we started with the pharmacological agent flupirtine that has properties to regulate cell excitability by opening the voltage-gated potassium channels KCNQ2/3 [13] and KCNQ/Kv7 channels in neurons [36]. The next pharmacological agent is diazoxide that activates ATP-sensitive potassium channels and is used in the treatment of hyperinsulinemia and hypoglycemia due to its ability to inhibit insulin release [2]. The last pharmacological agent is chlorzoxazone (Chl), an activator of calcium-activated potassium channels and an FDA-approved treatment for muscle pain [19,20,21].

### 2.1. Prefrontal Cortex

We studied the effect of potassium channel openers on the PFC because it is an important brain structure that participates in the regulation of self-control, decision making, and ingestive and social behaviors [27,28,29,30,31].

**Figure 1:**
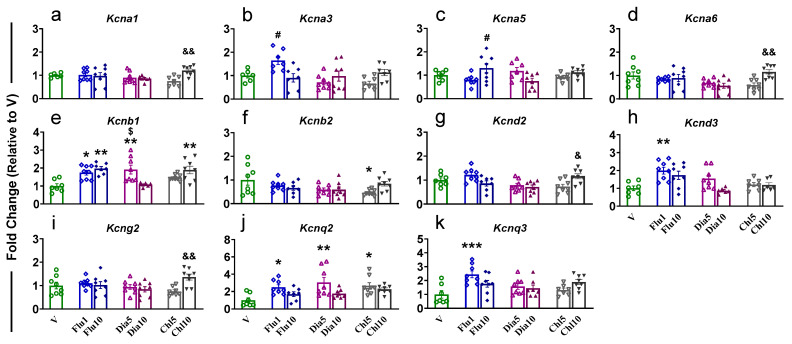
The effects of potassium agonist on the mRNA expression of voltage-gated potassium channels (**a**) *Kcna1*; (**b**) *Kcna3*; (**c**) *Kcna5*; (**d**) *Kcna6*; (**e**) *Kcnb1*; (**f**) *Kcnb2*; (**g**) *Kcnd2*; (**h**) *Kcnd3*; (**i**) *Kcng2*; (**j**) *Kcnq2;* and (**k**) *Kcnq3* in the PFC. Legend key: vehicle control, V; flupirtine (1 mg/kg), Flu1; flupirtine (10 mg/kg), Flu10; diazoxide (5 mg/kg), Dia5; diazoxide (10 mg/kg), Dia10; chlorzoxazone (5 mg/kg), Chl5; chlorzoxazone (10 mg/kg), Chl10. Key to statistics: * *p* < 0.05, ** *p* < 0.01, *** *p* < 0.001, comparison vehicle vs. individual potassium agonist; # *p* < 0.05, comparison between Flu1 and Flu10 taker groups; $ *p* < 0.05, comparison between Dia5 and Dia10 taker groups; & *p* < 0.05, && *p* < 0.01, comparison between Chl5 and Chl10 taker groups.

One-way ANOVA for *Kcna1* showed significant effects (F (6, 45) = 4.233, *p* = 0.0217), with dose-dependent increases in Chl10-treated rats compared to Chl5 (Figure 1a). Administration of Flu1 in rats resulted in a significant increase in *Kcna3* (F (6, 44) = 4.599, *p* = 0.0011) compared to Flu10 animals, whereas we saw an increased expression for *Kcna5* (F (6, 46) = 3.050, *p* = 0.0135) in Flu10-treated rats compared to Flu1 (Figure 1b and Figure 1c, respectively). *Kcna6* (F (6, 48) = 4.149, *p* = 0.0020) displayed significant increased expression in Chl10 rats compared to Chl5 animals (Figure 1d). *Kcnb1* (F (6, 45) = 6.225, *p* < 0.0001) expression was found to increase in Flu1-, Flu10-, Dia5-, and Chl10-exposed rats compared to V, Dia5, and Dia10, respectively, with increased levels of Dia5 vs. Dia10 (Figure 1e). Rats administered with Chl5 revealed a significant (F (6, 48) = 2.437, *p* = 0.0388) decrease in the level of *Kcnb2* in the PFC versus V (Figure 1f), while we saw a substantial (F (6, 47) = 4.814, *p* = 0.0007) increase in expression of *Kcnd2* in Chl10 animals compared to Chl5 (Figure 1g). *Kcnd3* expression was found to be significantly (F (6, 43) = 5.385, *p* = 0.0003) increased in Flu1 rats when compared to V (Figure 1h). Chl10-treated rats displayed higher mRNA levels of *Kcng2* than Chl5-treated rats (Figure 1i). Interestingly, only rats administered with the low doses of Flu1, Dia5, and Chl5 showed a significant increase (F (6, 46) = 3.983, *p* = 0.0027) in the expression of *Kcnq2* when compared to V (Figure 1j); no changes were seen in high-dose-treated rats. Finally, only Flu1-administered rats revealed increased (F (6, 46) = 4.345, *p* = 0.0015) expression of *Kcnq3* in the PFC compared to V animals (Figure 1k). We did not see any changes in the expression for *Kcna2*, *Kcna4*, *Kcnb3*, *Kcnd1*, *Kcng1*, *Kcng3, Kcng4*, *Kcnq1*, and *Kcnq4* in the PFC of rats that received Flu, Dia, and Chl (Supplementary Figure S1).

### 2.2. Nucleus Accumbens

We studied the NAc for its involvement in decision making, rewarding, and aversive responses [32,37,38]. Furthermore, inactivation of the voltage-gated potassium channel Kcna4/Kv1.4 in the NAc was found to be inversely proportional to motivation of natural reward [39].

**Figure 2:**
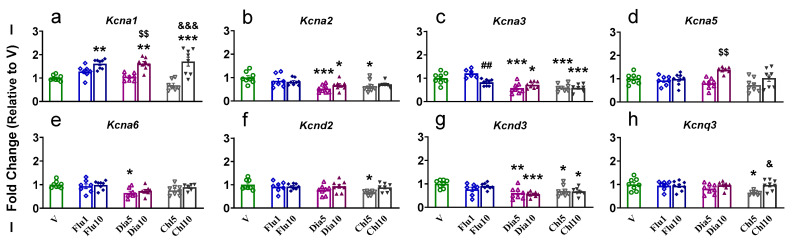
The effects of potassium agonist on the mRNA expression of voltage-gated potassium channels (**a**) *Kcna1*; (**b**) *Kcna2*; (**c**) *Kcna3*; (**d**) *Kcna5*; (**e**) *Kcna6*; (**f**) *Kcnd2*; (**g**) *Kcnd3*; and (**h**) *Kcnq3* in the NAc. Legend key: vehicle control, V; flupirtine (1 mg/kg), Flu1; flupirtine (10 mg/kg), Flu10; diazoxide (5 mg/kg), Dia5; diazoxide (10 mg/kg), Dia10; chlorzoxazone (5 mg/kg), Chl5; chlorzoxazone (10 mg/kg), Chl10. Key to statistics: * *p* < 0.05, ** *p* < 0.01, *** *p* < 0.001, comparison vehicle vs. individual potassium agonist; ## *p* < 0.01, comparison between Flu1 and Flu10 taker groups; $$ *p* < 0.01, comparison between Dia5 and Dia10 taker groups; & *p* < 0.05, &&& *p* < 0.001, comparison between Chl5 and Chl10 taker groups.

Rats administrated with Flu10, Dia10, and Chl10 exhibited a significantly (F (6, 47) = 13.87, *p* < 0.0001) higher mRNA level for *Kcna1* in their NAc compared to V (Figure 2a). Moreover, we also observed increased expression of *Kcna1* in Dia10- and Chl10-treated rats when compared to Dia5 and Chl5 animals, respectively (Figure 2a). *Kcna2* mRNA levels were found to be decreased (F (6, 46) = 4.595, *p* = 0.0010) in the Dia5-, Dia10-, and Chl5-treated rats compared to V (Figure 2b). Likewise, expression of *Kcna3* was also found to be lower (F (6, 46) = 12.38, *p* < 0.0001) in the Dia5-, Dia10-, Chl5-, and Chl10-administered rats compared to V and only in Flu10 vs. Flu1 (Figure 2c). Rats that received Dia10 injection showed significantly (F (6, 47) = 4.870, *p* = 0.0006) higher *Kcna5* mRNA levels compared to Dia5 (Figure 2d), while expression of *Kcna6* was found to be significantly (F (6, 46) = 3.869, *p* = 0.0033) reduced in Dia5 animals vs. V (Figure 2e). We found decreased expression of *Kcnd2* (F (6, 47) = 2.661, *p* = 0.0264) in Chl5 (Figure 2f) and *Kcnd3* (F (6, 47) = 5.556, *p* = 0.0002) in Dia5, Dia10, Chl5, and Chl10 compared to V (Figure 2g). *Kcnq3* expression also revealed a significant reduction (F (6, 46) = 2.987, *p* = 0.0151) in Chl5-treated rats compared to V and Chl10 (Figure 2h). There were no significant changes in *Kcna4*, *Kcnb1-3*, *Kcnd1*, *Kcng1*, *Kcng2*, *Kcng3*, *Kcng4*, *Kcnq1*, *Kcnq2*, and *Kcnq4* in the NAc (Supplementary Figure S2). Of note, we saw more downward decreases in the Kv in all treatments, and this may suggest that the K^+^ Ag tested may blunt the gene activation in the NAc.

### 2.3. Dorsal Striatum

The dSTR is one of the important brain regions involved in habit formation [33], reward [40], and decision making [41]. It is also known to play a crucial role in neurological and psychiatric disorders, including seizures [42]; epilepsy [43]; schizophrenia (for a review, see [44]); and pain transmission (for a review, see [45]). This led us to investigate the effects of potassium channel opener administration on the dSTR.

**Figure 3:**
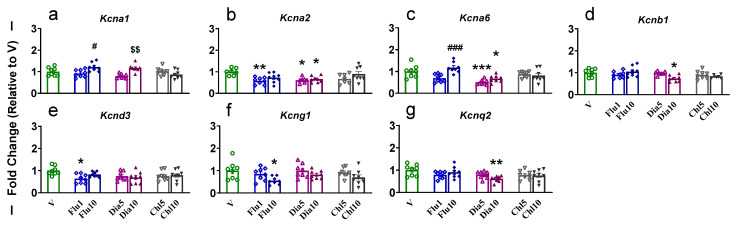
The effects of potassium agonist on the mRNA expression of voltage-gated potassium channels (**a**) *Kcna1*; (**b**) *Kcna2*; (**c**) *Kcna6*; (**d**) *Kcnb1*; (**e**) *Kcnd3*; (**f**) *Kcng1*; and (**g**) *Kcnq2* in the dSTR. Legend key: vehicle control, V; flupirtine (1 mg/kg), Flu1; flupirtine (10 mg/kg), Flu10; diazoxide (5 mg/kg), Dia5; diazoxide (10 mg/kg), Dia10; chlorzoxazone (5 mg/kg), Chl5; chlorzoxazone (10 mg/kg), Chl10. Key to statistics: * *p* < 0.05, ** *p* < 0.01, *** *p* < 0.001, comparison vehicle vs. individual potassium agonist; # *p* < 0.05, ### *p* < 0.001, comparison between Flu1 and Flu10 taker groups; $$ *p* < 0.01, comparison between Dia5 and Dia10 taker groups.

One-way ANOVA showed a significant increase (F (6, 49) = 5.996, *p* < 0.0001) in the expression of *Kcna1* in Flu10- and Dia10-treated rats when compared to Flu1 and Dia5, respectively (Figure 3a). The *Kcna2* mRNA was found to be significantly (F (6, 46) = 4.185, *p* = 0.0019) decreased in the Flu1, Dia5, and Dia10 groups compared to V (Figure 3b). The expression of *Kcna6* was found to increase (F (6, 49) = 8.875, *p* < 0.0001) in Flu10 compared to Flu1 and decrease in Dia5 and Dia10 compared to V (Figure 3c). Moreover, *Kcnb1* was also found to be significantly reduced (F (6, 46) = 3.313, *p* = 0.0086) in Dia10-administered rats when compared to V (Figure 3d). A similar decrease was also observed for *Kcnd3* (F (6, 49) = 2.322, *p* = 0.0472) in Flu1-treated rats (Figure 3e), *Kcng1* (F (6, 49) = 2.642, *p* = 0.0267) in Flu10-treated rats (Figure 3f), and *Kcnq2* (F (6, 49) = 2.766, *p* = 0.0214) in Dia10-treated rats (Figure 3g) compared to V. It is of note that Chl had no significant changes in the dSTR. There were no significant changes in *Kcna3*, *Kcna4, Kcna5*, *Kcnb2*, *Kcnb3*, *Kcnd1*, *Kcnd2*, *Kcng2*, *Kcng3*, *Kcng4*, *Kcnq1*, and *Kcnq3*, *Kcnq4* in the dSTR (Supplementary Figure S3).

### 2.4. Hippocampus

The HIP is an important structure for learning and memory formation [34,35]. Moreover, multiple articles have shown that hippocampal tissue can be subdivided into dorsal and ventral hippocampus (dHIP and vHIP) [46,47,48,49,50,51,52]. Furthermore, lesions in the dHIP are found to effect spatial memory, whereas lesions in the vHIP alter stress responses and emotional behavior [48,50,51]. These above findings led us to separately study the effect of potassium channel openers on dHIP and vHIP.

#### 2.4.1. Dorsal Hippocampus

**Figure 4:**
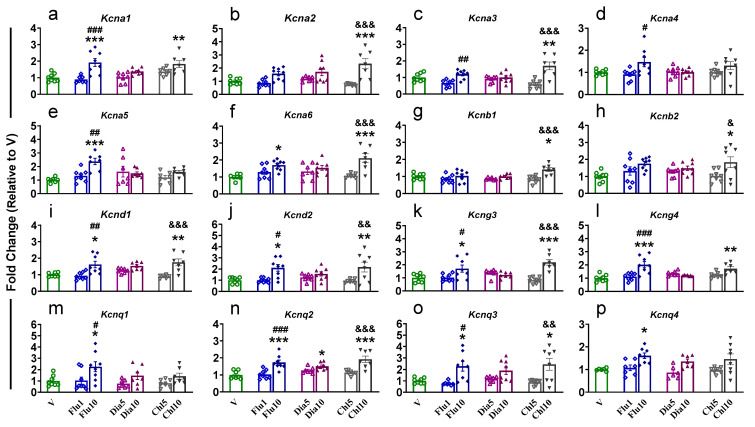
The effects of potassium agonist on the mRNA expression of voltage-gated potassium channels (**a**) *Kcna1*; (**b**) *Kcna2*; (**c**) *Kcna3*; (**d**) *Kcna4*; (**e**) *Kcna5*; (**f**) *Kcna6*; (**g**) *Kcnb1*; (**h**) *Kcnb2*; (**i**) *Kcnd1*; (**j**) *Kcnd2*; (**k**) *Kcng3*; (**l**) *Kcng4*; (**m**) *Kcnq1*; (**n**) *Kcnq2*; (**o**) *Kcnq3*; and (**p**) *Kcnq4* in the dHIP. Legend key: vehicle control, V; flupirtine (1mg/kg), Flu1; flupirtine (10 mg/kg), Flu10; diazoxide (5 mg/kg), Dia5; diazoxide (10 mg/kg), Dia10; chlorzoxazone (5 mg/kg), Chl5; chlorzoxazone (10 mg/kg), Chl10. Key to statistics: * *p* < 0.05, ** *p* < 0.01, *** *p* < 0.001, comparison vehicle vs. individual potassium agonist; # *p* < 0.05, ## *p* < 0.01, ### *p* < 0.001, comparison between Flu1 and Flu10 taker groups; & *p* < 0.05, && *p* < 0.01, &&& *p* < 0.001, comparison between Chl5 and Chl10 taker groups.

ANOVA for *Kcna1* showed a significantly (F (6, 47) = 8.457, *p* < 0.0001) elevated mRNA level in rats administered with a Flu10 and Chl10 dose when compared to V and in Flu10 animals compared to Flu1 (Figure 4a). Interestingly, compared to V and Chl5, Chl10-administered rats showed increased expression for *Kcna2* (F (6, 46) = 7.887, *p* < 0.0001, Figure 4b) and *Kcna3* (F (6, 46) = 9.289, *p* < 0.0001, Figure 4c), while Flu10-administered rats also showed higher expression of *Kcna3* (Figure 4c), *Kcna4* (F (6, 49) = 2.869, *p* = 0.0178, Figure 4d), and *Kcna5* (F (6, 49) = 2.869, *p* = 0.0178, Figure 4e) compared to Flu1. Moreover, *Kcna5* (Figure 4e) and *Kcna6* (F (6, 45) = 6.575, *p* < 0.0001, Figure 4f) mRNA levels were also higher in the dHIP of Flu10 compared to V. Additionally, Chl10 had higher levels for *Kcna6* (Figure 4f), *Kcnb1* (F (6, 45) = 5.338, *p* = 0.0003, Figure 4g), *Kcnb2* (F (6, 49) = 3.169, *p* = 0.0105, Figure 4h), *Kcnd1* (F (6, 43) = 7.546, *p* < 0.0001, Figure 4i), *Kcnd2* (F (6, 48) = 5.525, *p* = 0.0002, Figure 4j), *Kcng3* (F (6, 46) = 8.915, *p* < 0.0001, Figure 4k), *Kcnq2* (F (6, 46) = 10.44, *p* < 0.0001, Figure 4n)*, and Kcnq3* (F (6, 47) = 5.628, *p* = 0.0002, Figure 4o) compared to Chl5 and V. Likewise, the expression of *Kcnd1* (Figure 4i), *Kcnd2* (Figure 4j), *Kcng3* (Figure 4k), *Kcng4* (F (6, 45) = 8.866, *p* < 0.0001, Figure 4l), *Kcnq1* (F (6, 45) = 3.525, *p* = 0.0061, Figure 4m), *Kcnq2* (Figure 4n)*,* and *Kcnq3* (Figure 4o) was increased in Flu10 vs. Flu1 and V. A higher level of *Kcng4* (Figure 4l) was found in Chl 10, while Flu10 animals revealed increased expression for *Kcnq4* (F (6, 42) = 4.422, *p* = 0.0015, Figure 4p) compared to V. Finally, we observed a significant difference between Dia10 and V for *Kcnq2* (Figure 4n). There were no significant changes in *Kcnb3*, *Kcnd3*, and *Kcng1, Kcng2* in the dHIP (Supplementary Figure S4).

#### 2.4.2. Ventral Hippocampus

**Figure 5:**
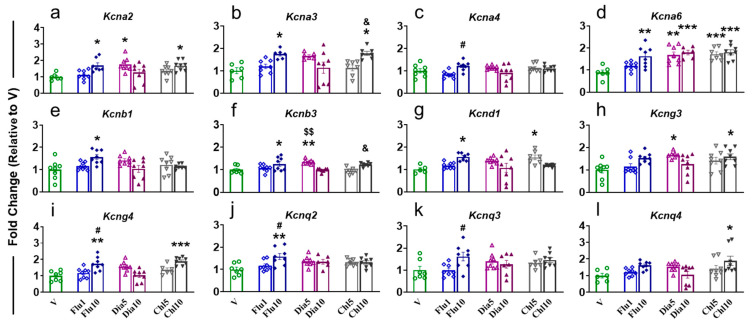
The effects of potassium agonist on the mRNA expression of voltage-gated potassium channels (**a**) *Kcna2*; (**b**) *Kcna3*; (**c**) *Kcna4*; (**d**) *Kcna6*; (**e**) *Kcnb1*; (**f**) *Kcnb3*; (**g**) *Kcnd1*; (**h**) *Kcng3*; (**i**) *Kcng4*; (**j**) *Kcnq2*; (**k**) *Kcnq3*; and (**l**) *Kcnq4* in the vHIP. Legend key: vehicle control, V; flupirtine (1 mg/kg), Flu1; flupirtine (10 mg/kg), Flu10; diazoxide (5 mg/kg), Dia5; diazoxide (10 mg/kg), Dia10; chlorzoxazone (5 mg/kg), Chl5; chlorzoxazone (10 mg/kg), Chl10. Key to statistics: * *p* < 0.05, ** *p* < 0.01, *** *p* < 0.001, comparison vehicle vs. individual potassium agonist; # *p* < 0.05, comparison between Flu1 and Flu10 taker groups; $$ *p* < 0.01, comparison between Dia5 and Dia10 taker groups, & *p* < 0.05, comparison between Chl5 and Chl10 taker groups.

In the vHIP, *Kcna2* was found to be significantly increased (F (6, 46) = 4.478, *p* = 0.0012) in Flu10-, Dia5-, and Chl10-treated rats compared to V (Figure 5a). *Kcna3* expression was increased (F (6, 41) = 5.029, *p* = 0.0006) in Flu10 and Chl10 animals compared to V (Figure 5b). Moreover, Chl10 rats also showed a higher level of Kcna3 compared to Chl5 (Figure 5b). Flu10 rats had a higher (F (6, 48) = 3.15, *p* = 0.011) level of *Kcna4* expression than Flu1 (Figure 5c), while expression of *Kcna6* was found to significantly increase (F (6, 44) = 7.553, *p* < 0.0001) in Flu10, Dia5, Dia10, CHhl5, and Chl10 when compared to V (Figure 5d). The mRNA level of *Kcnb1* was only found to be elevated (F (6, 48) = 3.321, *p* = 0.0081) in Flu10-injected rats compared to V (Figure 5e). Higher expression for *Kcnb3* (F (6, 45) = 6.641, *p* < 0.0001) was seen in rats treated with Flu10 and Dia5 versus V, and a dose-dependent increase in Dia5 and Chl10 was observed when compared to Dia10 and Chl5, respectively (Figure 5f). The level of *Kcnd1* was also elevated (F (6, 43) = 4.207, *p* = 0.0020) in Flu10 and Chl5 vs. V rats (Figure 5g). We also observed a higher (F (6, 48) = 3.715, *p* = 0.0041) mRNA level for *Kcng3* in Dia5- and Chl10-administered rats compared to V (Figure 5h). *Kcng4* expression was found to be significantly increased (F (6, 49) = 6.481, *p* < 0.0001) in Flu10 and Chl10 vs. V rats, and there was a dose-dependent increase in Flu10 compared to Flu1 animals (Figure 5i). Flu10-treated rats exhibited elevated levels for *Kcnq2* (F (6, 45) = 3.464, *p* = 0.0067, Figure 5j) and *Kcnq3* (F (6, 46) = 2.678, *p* = 0.0259, Figure 5k) in their vHIP compared to Flu1; moreover, for *Kcnq2*, Flu10 rats also revealed higher mRNA levels than V (Figure 5j). Only Chl10 animals displayed a higher mRNA level for *Kcnq4* (F (6, 46) = 2.678, *p* = 0.0259) when compared to V rats (Figure 5l). There were no significant changes in *Kcna1*, *Kcna5*, *Kcnb2*, *Kcnd2*, *Kcnd3*, *Kcng1*, *Kcng2*, and *Kcnq1* in the vHIP (Supplementary Figure S5).

Regardless of the gene expression of the other tissues, we found the Kv gene (*Kcna1*, *Kcna5*, *Kcnb1*, *Kcnb2*, *Kcnd2*, *Kcnq1*, and *Kcnq3*) expression changes only in the dHIP and not in the vHIP, whereas *Kcnb3* was only changed in the vHIP when compared to the dHIP. Our results agree with Lee et al., 2017, where they also observed differential gene expression in the d- or vHIP in all developmental stages (juvenile, adolescent, and adult) of SD rats [49]. Our current data along with those of other investigators support the notion of studying the dorsal and ventral hippocampus as separate neuronal structures [53].

## 3. Discussion

In the present study, we examined the effects produced by administration of potassium channel openers, flupirtine and FDA-approved diazoxide and chlorzoxazone, on the expression profile of voltage-gate potassium channels in the mesocorticolimbic and nigrostriatal dopaminergic systems (PFC, NAc, dHIP, vHIP, and dSTR). Our results showed that (i) treatment with flupirtine, diazoxide, and chlorzoxazone caused an alteration in the expression pattern of voltage-gated potassium channels; (ii) each brain region had its own unique gene expression signature; (iii) of interest, Chl caused no effect in the dSTR; and (iv) the effects of potassium channel openers showed differential Kv expression between the dHIP and vHIP (please see Table 1).

### 3.1. Diazoxide

Diazoxide is FDA approved for the treatment of hypoglycemia and acute hypertension [2]. Diazoxide has also been proven effective in animal models for stroke [5]. Administration of diazoxide was found to promote myelination in neurons and thereby attenuate brain injury in the animal model of hypoxia [54]. Additionally, administration of diazoxide has also been reported to reduce NMDA-induced hippocampal neurodegeneration by decreasing neuronal loss and microglia activation [55]. We found that administration of Dia resulted in altered expression of *Kcna6* in the NAc, dSTR, and vHIP, while *Kcnq2* mRNA levels were found to be affected in the PFC, dSTR, and dHIP.

When we talk about *Kcna6*, its increased expression has been reported in sciatic nerve injury and has been associated with neuropathic pain and Morton’s neuroma [56]. Using a knockout mice model, Peck et al. (2021) also showed that *Kcna6* plays an important role in the progression of acute and neuropathic pain [57]. Moreover, KCNQ2 has also been reported to play a critical role in modulating susceptibility to seizures and epilepsy [58]. From our finding along with others, it can be inferred that Dia may bring its effect via altering the expression of *Kcna6* and *Kcnq2* in multiple brain regions.

### 3.2. Chlorzoxazone

Chlorzoxazone is a calcium/sodium -K^+^ channel activator, but we want to check how this drug affects Kv channel expression in the brain. Clinical studies have shown that Chl is a skeletal muscle relaxant [59] and has been found to activate the large-conductance calcium-dependent potassium channels/KCNMA1 [60]. In the present study, administration of Chl resulted in differential expression of Kv in multiple brain regions of the rats. Specifically, *Kcna2* and *Kcna3* changed their expression in the NAc, dHIP, and vHIP, while we observed an increased mRNA level for *Kcna6* in the PFC, dHIP, and vHIP. From a therapeutic point of view, administration of Chl was reported to restore impaired BKCa functionality, resulting in positive behavioral and cogitative outcomes in a mice model for Fragile X syndrome [61,62], Williams–Beuren syndrome [63], spinocerebellar ataxia [64,65], and alcohol intake [66]. The positive outcome seen in various disease models might be associated with the alteration in the expression of *Kcna2*, *Kcna3*, and *Kcna6* after Chl administration. Further, it has been reported that mutations and abnormal functions of KCNA2 in humans can lead to different neurological diseases that include epilepsy, autism spectrum disorder, pain, movement disorders [8,67], and temporal lobe epilepsy [18]. Moreover, complete loss-of-function (LOF) in the p.H310R variant in KCNA2 results in epilepsy, while a gain-of-function (GOF) p.H310Y variant was found to be involved in developmental delay in children [3]. Additionally, KCNA3 de novo missense variants have been associated with the development of epileptic encephalopathies in humans [68]. Adding to the story, GOF in KCNA6 was found to be associated with early infantile epileptic phenotypes and neurodevelopmental anomalies [69]. Furthermore, Chl administration led to more profound changes in the expression of voltage-gated potassium channels in the PFC, NAc, and HIP and not in the dSTR of rats. This suggests that the mechanism of action of Chl is via regulation of the expression of Kv in the mesocorticolimbic dopaminergic system.

### 3.3. Flupirtine

Flupirtine was marketed as a non-opioid analgesic and was used for the treatment of acute and chronic pain for decades in Europe [1]. Flu regulates cell excitability by opening the voltage-gated potassium channels KCNQ2/3 and was a promising drug to treat hyperexcitability [24,25,26]. However, Flu caused a series of side effects due to its poor target selectivity and was removed from clinical use [26]. We used Flu because of its pharmacologic capabilities and limited side effects in the single acute use as per the European Medicines Agency [70]. The rats that received Flu treatment revealed alterations in expression for *Kcna1* in the NAc, dSTR, and dHIP; *Kcna3* in the PFC, NAc, dHIP, and vHIP; *Kcna6* in the dSTR, dHIP, and vHIP; and *Kcnq2* and *Kcnq3* in the PFC, dHIP, and vHIP.

Mutations and abnormal functions of KCNA1/Kv1.1 channels can lead to changes in neuronal excitability, leading to epilepsy and other neurological disorders in humans [8,71]. Even more noteworthy, epilepsy can occur with mutations/changes in KCNA1 by both LOF and GOF [72]. It was reported that 50% of KCNA1/Kv1.1 null mice died with seizures between the third and fifth week of their life, even though having normal cardiac rhythm [73].

KCNQ2/Kv7.2 and KCNQ3/Kv7.3 can form heterotetramers that limit neuronal hyperexcitability and firing frequency [74,75,76,77]. KCNQ2 and KCNQ3 play a critical role in modulating susceptibility to seizures, and their mutations cause heterogeneous epilepsy phenotypes [58]. Moreover, GOF of KCNQ2 and KCNQ3 leads to neurodevelopmental disorders with no established treatments, while LOF (KCNQ2 and KCNQ3) causes neuronal hyperexcitability or neonatal seizures [78,79]. Interestingly, either KCNQ2 GOF or LOF (p.V175L mutation) is found to produce early-onset epileptic encephalopathy in children [80,81,82]. The normalization of the potassium current may serve as a treatment for the epilepsy phenotypes.

Additionally, administration of Flu is reported to exert protective effects on CRS-induced cognitive impairment [83] and reduce cocaine conditioned place preference behavior [84] and neuronal damage in a rat model of global ischemia [85]. Flu is also reported to have antiparkinsonian action [86,87]. These positive outcomes might be associated with the alteration in the expression of potassium channels after Flu administration. Given the plethora of Kv gene expression changes in Flu, we propose the mechanisms of action are rooted in activation or deactivation of one or multiple combinations of these potassium channel genes: *Kcna1*, *Kcna3*, *Kcna6*, *Kcnq2*, and *Kcnq3*.

## 4. Materials and Methods

### 4.1. Potassium Channel Openers

The stock solutions of flupirtine maleate (R&D Systems, Minneapolis, MN, USA) and FDA-approved drugs diazoxide (Sigma-Aldrich, Billerica, MA, USA) and chlorzoxazone (Sigma-Aldrich) were prepared by dissolving in the vehicle (V) composed with Cremophor^®^ EL (15% final volume, Sigma-Aldrich), ethanol (45–60% final volume, The Warner-Graham Co., Cockeysville, MD, USA) [88], and final volume adjusted with saline (0.9% sodium chloride, Hospira, Inc., Lake Forest, IL, USA).

### 4.2. Ethical Approval

All animal procedures were approved by the National Institute of Drug Abuse Animal Care and Use Committee and conducted per the Guide for the Care and Use of Laboratory Animals (ISBN 0-309-05377-3). The approved protocol number for these experiments was 23-MNPB-9, Approval Date: 13 February 2024.

### 4.3. Animals and Potassium Channel Openers

Fifty-six male Sprague Dawley (SD) rats (Charles River Laboratories, Kingston, NY, USA) weighing 350–500 g were group housed under 12 h light/dark cycle with free access to food and water. We used 8 rats per group for intraperitoneal (i.p.) administration of V and different doses of potassium channel openers as per pervious papers: Flu (1 and 10 mg/kg) [86,87], Dia (5 and 10 mg/kg) [89,90], and Chl (5 and 10 mg/kg) [61,62,66], with all injections less than 0.5 mL.

### 4.4. Tissue Collection and RNA Extraction

To measure potential region-specific differences in gene expression, rats were euthanized 2 h after injection. Monitoring at this time helps assess the peak concentration and initial biological effects of the drug or compound. This timing is crucial for evaluating the efficacy, pharmacokinetics, and potential side effects of the IP-administered agent. Prefrontal cortex (A/P + 2.7 to +1.7 mm bregma, M/L 0 to +4 mm, D/V + 7 to +9 mm), nucleus accumbens (A/P + 2.7 to +0.7 mm bregma, M/L + 0.6 to +2.2 mm, D/V + 5.6 to +7.6 mm), dorsal striatum (A/P + 2 to −2 mm bregma, M/L ± 2 to 5 mm, D/V − 3 to −6 mm), dorsal hippocampus (A/P − 2.8 mm bregma, M/L − 5.3 mm, D/V − 2.0 mm), and ventral hippocampus (A/P − 5.3 mm bregma, M/L − 6.72 mm, D/V − 7.0 mm) tissues were dissected from the brains, immediately snap-frozen on dry ice, and stored at −80 °C. Total RNA was then isolated using Qiagen RNeasy Mini kits (Qiagen, Germantown, MD, USA) and treated with Qiagen RNase-free DNase kits following the manufacturer’s protocol. Finally, we used a NanoDrop 2000 spectrophotometer (Thermo Fisher Scientific, Waltham, MA, USA) to assess RNA concentrations.

### 4.5. Quantitative RT-PCR

One-half microgram (0.5 µg) of total RNA was reverse transcribed to cDNA using oligo dT primer from the Advantage RT for PCR kit (Clontech, Takara Bio, Mountain View, CA, USA). The qPCR primers were acquired from the Sequencing Facility at Johns Hopkins University (Baltimore, MD, USA) and IDT (Integrated DNA Technologies, Inc., Coralville, IA, USA). The qRT-PCR reactions were performed in a LightCycler 480 II instrument (Roche, Indianapolis, IN, USA) using LUNA (New England Biolabs, Inc., Ipswich, MA, USA). Data were normalized using the corresponding reference mRNA levels of *B2M*, *Rn18s*, *Rps5*, *Tbp*, *Tubb2b*, or *UBC*. The results are reported as fold changes calculated as the ratios of normalized gene expression data for K^+^ Ag-treated groups (at various dosages) in comparison to the vehicle group (V). Primer sequences for voltage-gated potassium channels (Kv channels) used in this study are listed in Table 2.

### 4.6. Statistical Analyses

Throughout this study, all treatment groups underwent the same paradigm. The qPCR results of relative mRNA abundance were analyzed by one-way ANOVA, followed by Tukey’s multiple comparisons post hoc test using GraphPad Prism 10 (San Diego, CA, USA). For all analyses, the null hypothesis was rejected at *p* ≤ 0.05.

## 5. Conclusions

Our study is the first to describe the effects of potassium channel openers (Flu, Dia, and Chl) on the mesocorticolimbic and nigrostriatal dopaminergic systems (PFC, NAc, dSTR, dHIP, and vHIP). In conclusion, we observed that administration of Dia altered expression of *Kcna6* and *Kcnq2*; Chl treatment changed mRNA levels of *Kcna2*, *Kcna3*, and *Kcna6*; and Flu injection resulted in transcript level variations in *Kcna1*, *Kcna3*, *Kcna6*, *Kcnq2*, and *Kcnq3* in the various brain regions tested. Of note, expression of *Kcna6* was found to be affected in the rat brain in response to Dia, Chl, and Flu treatment. We demonstrated a new mechanism of action for these potassium channel openers besides their known functions. They cause targeted increases and decreases in potassium channel genes that could aid in therapeutic outcomes. This is the first step, and additional research is necessary to understand to what extent these mechanisms might impact therapeutic interventions against neurological and psychiatric disorders.

Our findings provide critical and novel insights into the neuro-molecular consequences of potassium channel opener administration. However, a limitation of our study is that it was conducted exclusively in male rats. Future investigations should incorporate both male and female subjects, employ larger sample sizes, and include functional validation at the receptor level to comprehensively assess the potential neuro-molecular effects of potassium channel openers. Such an approach will enhance the translational relevance of our findings and support the potential repurposing of these drugs.

## Figures and Tables

**Table 1:** Summarized gene expression in different brain regions.

	PFC						NAc						dSTR						dHIP						vHIP					
Gene	Flu 1	Flu 10	Dia5	Dia10	Chl5	Chl10	Flu 1	Flu 10	Dia5	Dia10	Chl5	Chl10	Flu 1	Flu 10	Dia5	Dia10	Chl5	Chl10	Flu 1	Flu 10	Dia5	Dia10	Chl5	Chl10	Flu 1	Flu 10	Dia5	Dia10	Chl5	Chl10
*Kcna1*	−	−	−	−	−	**↑** **d**	−	**↑** **a**	−	**↑** **ac**	−	**↑** **ad**	−	**↑** **b**	−	**↑** **c**	−	−	−	**↑** **ab**	−	−	−	**↑** **a**	−	−	−	−	−	−
*Kcna2*	−	−	−	−	−	−	−	−	**↓** **a**	**↓** **a**	**↓** **a**	−	**↓** **a**	−	**↓** **a**	**↓** **a**	−	−	−	−	−	−	−	**↑** **ad**	−	**↑** **a**	**↑** **a**	−	−	**↑** **a**
*Kcna3*	**↑** **b**	−	−	−	−	−	−	**↓** **b**	**↓** **a**	**↓** **a**	**↓** **a**	**↓** **a**	−	−	−	−	−	−	−	**↓** **b**	−	−	−	**↑** **ad**	−	**↑** **a**	−	−	−	**↑** **ad**
*Kcna4*	−	−	−	−	−	−	−	−	−	−	−	−	−	−	−	−	−	−	−	**↑** **b**	−	−	−	−	−	**↑** **b**	−	−	−	−
*Kcna5*	**↑** **b**	−	−	−	−	−	−	−	−	**↑** **c**	−	−	−	−	−	−	−	−	−	**↑** **ab**	−	−	−	−	−	−	−	−	−	−
*Kcna6*	−	−	−	−	−	**↑** **d**	−	−	**↓** **a**	−	−	−	−	**↑** **b**	**↓** **a**	**↓** **a**	−	−	−	**↑** **a**	−	−	−	**↑** **ad**	−	**↑** **a**	**↑** **a**	**↑** **a**	**↑** **a**	**↑** **a**
*Kcnb1*	**↑** **a**	**↑** **a**	**↑** **ac**	−	−	**↑** **a**	−	−	−	−	−	−	−	−	−	**↓** **a**	−	−	−	−	−	−	−	**↑** **ad**	−	**↑** **a**	−	−	−	−
*Kcnb2*	−	−	−	−	**↓** **a**	−	−	−	−	−	−	−	−	−	−	−	−	−	−	−	−	−	−	**↑** **ad**	−	−	−	−	−	−
*Kcnb3*	−	−	−	−	−	−	−	−	−	−	−	−	−	−	−	−	−	−	−	−	−	−	−	−	−	**↑** **a**	**↑** **ac**	−	−	**↑** **d**
*Kcnd1*	−	−	−	−	−	−	−	−	−	−	−	−	−	−	−	−	−	−	−	**↑** **ab**	−	−	−	**↑** **ad**	−	**↑** **a**	−	−	**↑** **a**	−
*Kcnd2*	−	−	−	−	−	**↑** **d**	−	−	−	−	**↓** **a**	−	−	−	−	−	−	−	−	**↑** **ab**	−	−	−	**↑** **ad**	−	−	−	−	−	−
*Kcnd3*	**↑** **a**	−	−	−	−	−	−	−	**↓** **a**	**↓** **a**	**↓** **a**	**↓** **a**	**↓** **a**	−	−	−	−	−	−	−	−	−	−	−	−	−	−	−	−	−
*Kcng1*	−	−	−	−	−	−	−	−	−	−	−	−	−	**↓** **a**	−	−	−	−	−	−	−	−	−	−	−	−	−	−	−	−
*Kcng2*	−	−	−	−	−	**↑** **d**	−	−	−	−	−	−	−		−	−	−	−	−	−	−	−	−	−	−	−	−	−	−	−
*Kcng3*	−	−	−	−	−	−	−	−	−	−	−	−	−	−	−	−	−	−	−	**↑** **ab**	−	−	−	**↑** **ad**	−	−	**↑** **a**	−	−	**↑** **a**
*Kcng4*	−	−	−	−	−	−	−	−	−	−	−	−	−	−	−	−	−	−	−	**↑** **ab**	−	−	−	**↑** **a**	−	**↑** **ab**	−	−	−	**↑** **a**
*Kcnq1*	−	−	−	−	−	−	−	−	−	−	−	−	−	−	−	−	−	−	−	**↑** **ab**	−	−	−	−	−	−	−	−	−	−
*Kcnq2*	**↑** **a**	−	**↑** **a**	−	**↑** **a**	−	−	−	−	−	−	−	−	−	−	**↓** **a**	−	−	−	**↑** **ab**	−	**↑** **a**	−	**↑** **ad**	−	**↑** **ab**	−	−	−	−
*Kcnq3*	**↑** **a**	−	−	−	−	−	−	−	−	−	**↓** **ad**	−	−	−	−	−	−	−	−	**↑** **ab**	−	−	−	**↑** **ad**	−	**↑** **b**	−	−	−	−
*Kcnq4*	−	−	−	−	−	−	−	−	−	−	−	−	−	−	−	−	−	−	−	**↑** **a**	−	−	−	−	−	−	−	−	−	**↑** **a**

Legend Key: Flu1, Flupirtine (1 mg/kg); Flu10, Flupirtine (10 mg/kg); Dia5, Diazoxide (5 mg/kg); Dia10, Diazoxide (10 mg/kg); Chl5, Chlorzoxazone (5 mg/kg); Chl10, Chlorzoxazone (10 mg/kg). Key to statistics: ↑ Up arrow, Significant upregulated; **↓** Down arrow, Significant downregulated; − hyphen, Not significant. Changes: a, comparison vehicle vs. individual potassium agonist; b, Flu10 vs. Flu1; c, Dia10 vs. Dia5; d, Chl10 vs. Chl5.

**Table 2:** List of RT-PCR primers and sequences.

Primers	Seq Up	Seq Down
*Kcna1*	TTG GTA AGG GTG TTC AGA AT	GCA AAG TAC ACT GCA CTA GA
*Kcna2*	TCT CCA TGA CAA CTG TAG GCT ATG	GAC TGG TAA GGC AAT GGT TAA CAC
*Kcna3*	TCA TCT TCT GCT TGG AGA CA	TAT TTC TGG AGA AGG TGG CTT TA
*Kcna4*	CAT GAC AAC TGT GGG CTA CGG	CGG GCA AAG CAA TGG TTA AGA
*Kcna5*	TTC TCT AGT ATC CCA GAT GC	CCC GAT GAT AGA AGT AAT TAA AG
*Kcna6*	TAT GGA AGA GAT TCG CTT CTA	GAA CTC TCC GGA TAC TCA AA
*Kcnb1*	CTC CAT CTA CAC CAC AGC AAG T	CTG AAC TTG GGA CTG GTA CTC C
*Kcnb2*	CAC AAC TGT AGG CAA GAC ATT TA	TCC TGG GTT AGA ATG AAT TTC TG
*Kcnb3*	AGC TTA ATC CAA ATA GCA AGT G	AGC CAT ATA TTA GGA CAA GGG
*Kcnd1*	GAA TCT TCA AGT TCT CCA GGC A	AAA GAT GAT GAT AGC CAT GGT
*Kcnd2*	TTG TGA ATG AGC ACA ATG AAA	ATT CAA CTG GCA CAT TAT GTC
*Kcnd3*	TCT TGT GGA TGA TCC CCT GTT G	GGG TAG TTC TGC ATT GAG CTC T
*Kcng1*	AAA GGA TCT GTG TCT CTT AGT	CTT AAA GGT CTG TCT GTT TGC
*Kcng2*	GTA GCC TGG AGG AGA TCG CAA	GGA ATT TCT GGG ACT CAA TTT T
*Kcng3*	CTC TCC GCT GAG TTC CTG AAT T	CCC AGG GAG AAA CAC GTG AAT A
*Kcng4*	TGT CCA CAT ATC CAT GTG TTC	GGT CAC TTT ATT TCA GAT TCG TC
*Kcnq1*	GTG ATG TTG ACC ACT TCC GAA TAC	TCA CTT TAG GGG AGA AGT TGT CAG
*Kcnq2*	CTC TAC TCT GGT GAG GAA TAA TC	AAC CGA GGG CTC TAT TAT ATC
*Kcnq3*	CCA GGA TGA GGA ATG CAA ATT AGT C	TCA GGA GGA GTA AAA ATG GGT GAT T
*Kcnq4*	ATA GGC AGA AAC ACT TTG AGA	TAG TAA TAC CAG GTG GCT GTC
*Rps5*	CTC CAT GAT GAT GCA CGG	CCA ATG CGT GTG GAG TC
*Ubc*	CAA CAT CCA GAA GGA GTC	GTA CGA GTA TCT TCC TGT TT
*Tbp*	ACT CTT CCA TTC TCA AAC TCT A	GTC AAG TTT ACA GCC AAG AT
*Tubb2b*	TAC AAC GAA GCA ACT GGT AAT	AGC TTT CTG ACT CCT TCC TAA
*Rn18s*	GCG CAA ATT ACC CAC T	ATC CAA CTA CGA GCT T
*B2m*	GAT CTT TCT GGT GCT TGT	AGC TCA ATT TCT ATT TGA GGT

## Data Availability

Data is available upon request.

## Supplementary Materials

The following supporting information can be downloaded at: https://www.mdpi.com/article/10.3390/ph18101446/s1, Figure S1: The effects of potassium agonist on the mRNA expression of voltage-gated potassium channels in the PFC; Figure S2: The effects of potassium agonist on the mRNA expression of voltage-gated potassium channels in the NAc; Figure S3: The effects of potassium agonist on the mRNA expression of voltage-gated potassium channels in the dSTR; Figure S4: The effects of potassium agonist on the mRNA expression of voltage-gated potassium channels in the dHIP; Figure S5: The effects of potassium agonist on the mRNA expression of voltage-gated potassium channels in the vHIP.

## Abbreviations

K^+^ channel, potassium channel;	potassium channel
K^+^ Ag	potassium channel opener
V	vehicle control
Flu	flupirtine
Dia	diazoxide
Chl	chlorzoxazone
PFC	prefrontal cortex
NAc	nucleus accumbens
dSTR	dorsal striatum
HIP	hippocampus
dHIP	dorsal hippocampus
vHIP	ventral hippocampus
SD	Sprague Dawley
PCs	pyramidal cells
KO	knockout
LOF	loss-of-function
GOF	gain-of-function
DA	dopamine
ANOVA	analysis of variance
qRT-PCR	quantitative reverse transcriptase polymerase reaction
Kv	voltage-gated potassium channel
Kca	calcium-activated potassium channel
K2p	two/tandem-pore domain potassium channel
Kir	inwardly rectifying potassium channel
LC-MS	liquid chromatography–mass spectrometry
B2m	beta-2 microglobulin
Rps5	ribosomal protein S5
Tbp	ATA box binding protein
Rn18s	18S ribosomal RNA
Tubb2b	tubulin, beta 2B class IIb
Ubc	ubiquitin C
