# Supplementary material for: Pharmacological Actions of Potassium Channel Openers on Voltage-Gated Potassium Channels
Source: Pharmaceuticals (Basel). 2025 Sep 26;18(10):1446. doi: 10.3390/ph18101446 (PMC12567514; doi:10.3390/ph18101446)
Supplement: Supplementary file 1 [file pharmaceuticals-18-01446-s001.zip › pharmaceuticals-3822751-supplementary.pdf]

Supplementary File

Pharmacological Actions of Potassium Channel Openers on Voltage Gated Potassium Channels

Michael T. McCoy, Bruce Ladenheim, Jean Lud Cadet and Atul P. Daiwile *

Molecular Neuropsychiatry Research Branch, NIDA Intramural Research Program, Baltimore, MD 21224, USA; mmccoy@intra.nida.nih.gov (M.T.M.); bnlgsp12@gmail.com (B.L.); jcadet@intra.nida.nih.gov (J.L.C.)

* Correspondence: atul.daiwile@nih.gov; Tel.: +1-667-312-5709

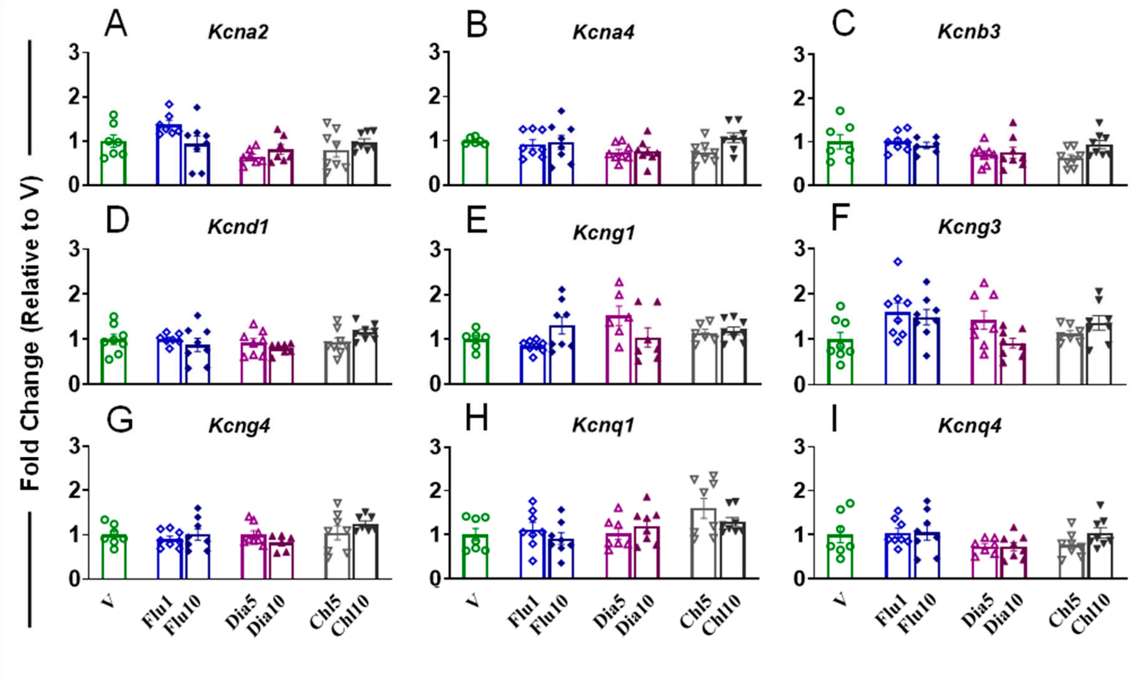

Figure S1. The effects of potassium agonist on the mRNA expression of voltage-gated potassium channels A) *Kcna2*; B) *Kcna4*; C) *Kcnb3*; D) *Kcnd1*; E) *Kcng1*; F) *Kcng3*; G) *Kcng4*; H) *Kcnq1*; and I) *Kcnq4* in the PFC. Legend Key; vehicle control, V; flupirtine (1 mg/kg), Flu1; flupirtine (10 mg/kg), Flu10; diazoxide (5 mg/kg), Dia5; diazoxide (10 mg/kg), Dia10; chlorzoxazone (5mg/kg), Chl5; chlorzoxazone (10mg/kg), Chl10. Key to statistics -- no change.

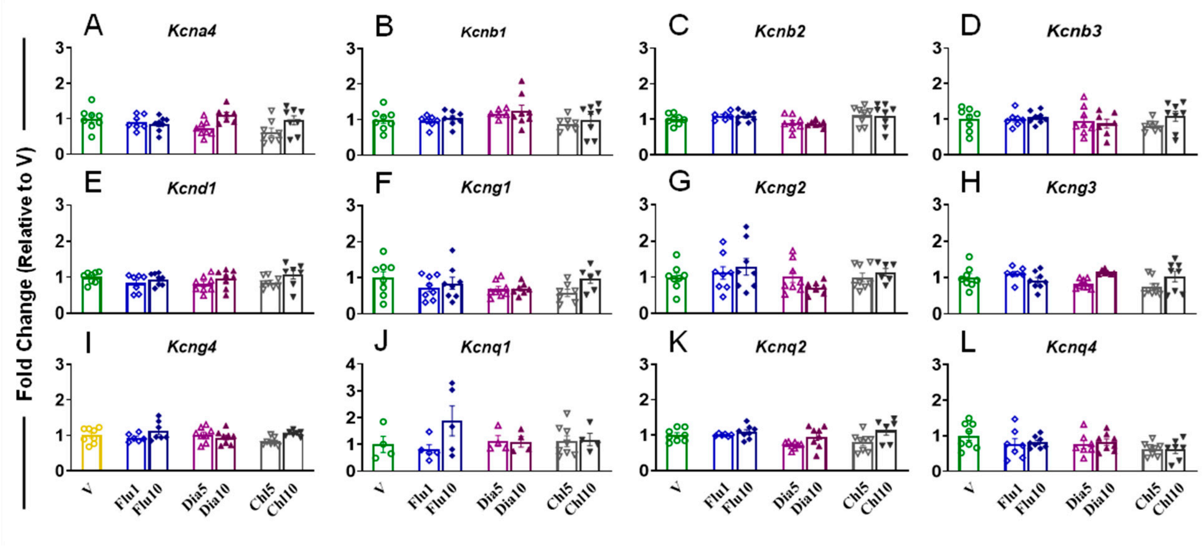

Figure S2. The effects of potassium agonist on the mRNA expression of voltage-gated potassium channels A) *Kcna4*; B) *Kcnb1*; C) *Kcnb2*; D) *Kcnb3*; E) *Kcnd1*; F) *Kcng1*; G) *Kcng2*; H) *Kcng3*; I) *Kcng4*; J*) Kcnq1*; K*) Kcnq2*; and L) *Kcnq4* in the NAc. Legend Key; vehicle control, V; flupirtine (1 mg/kg), Flu1; flupirtine (10 mg/kg), Flu10; diazoxide (5 mg/kg), Dia5; diazoxide (10 mg/kg), Dia10; chlorzoxazone (5 mg/kg), Chl5; chlorzoxazone (10 mg/kg), Chl10. Key to statistics -- no change.

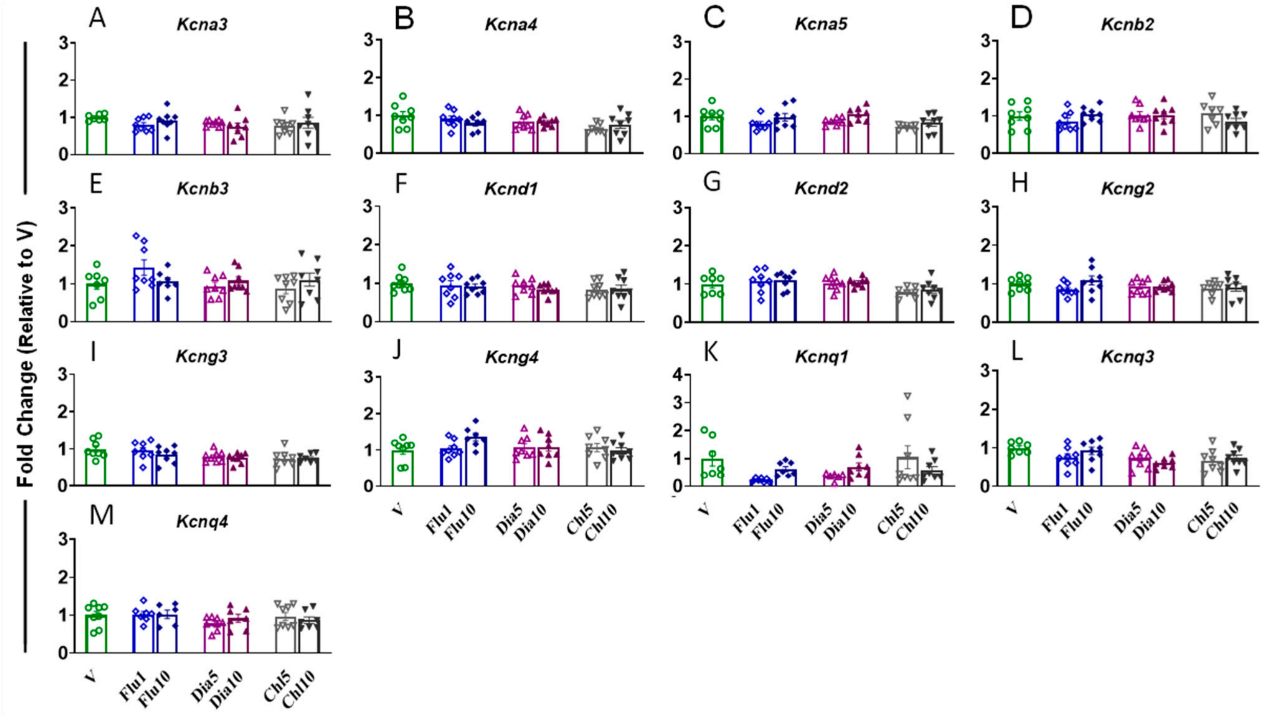

Figure S3. The effects of potassium agonist on the mRNA expression of voltage-gated potassium channels A) *Kcna3*; B) *Kcna4*; C) *Kcna5*; D) *Kcnb2*; E) *Kcnb3*; F) *Kcnd1*; G) *Kcnd2*; H) *Kcng2*; I) *Kcng3*; J*) Kcng4;* K*) Kcnq1;* L) *Kcnq3;* and M*) Kcnq4* in the dSTR. Legend Key; vehicle control, V; flupirtine (1 mg/kg), Flu1; flupirtine (10 mg/kg), Flu10;

diazoxide (5 mg/kg), Dia5; diazoxide (10 mg/kg), Dia10; chlorzoxazone (5 mg/kg), Chl5; chlorzoxazone (10mg/kg), Chl10. Key to statistics -- no change.

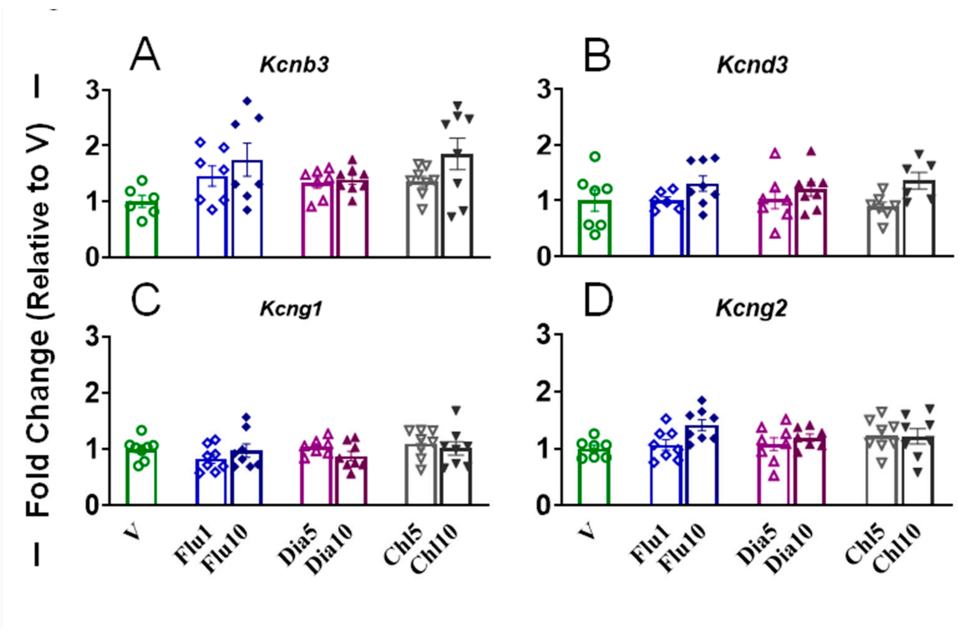

Figure S4. The effects of potassium agonist on the mRNA expression of voltage-gated potassium channels A) *Kcnb3*; B) *Kcnd3*; C) *Kcng1*; and D) *Kcnq2* in the dHIP. Legend Key; vehicle control, V; flupirtine (1 mg/kg), Flu1; flupirtine (10 mg/kg), Flu10; diazoxide (5 mg/kg), Dia5; diazoxide (10 mg/kg), Dia10; chlorzoxazone (5 mg/kg), Chl5; chlorzoxazone (10 mg/kg), Chl10. Key to statistics -- no change.

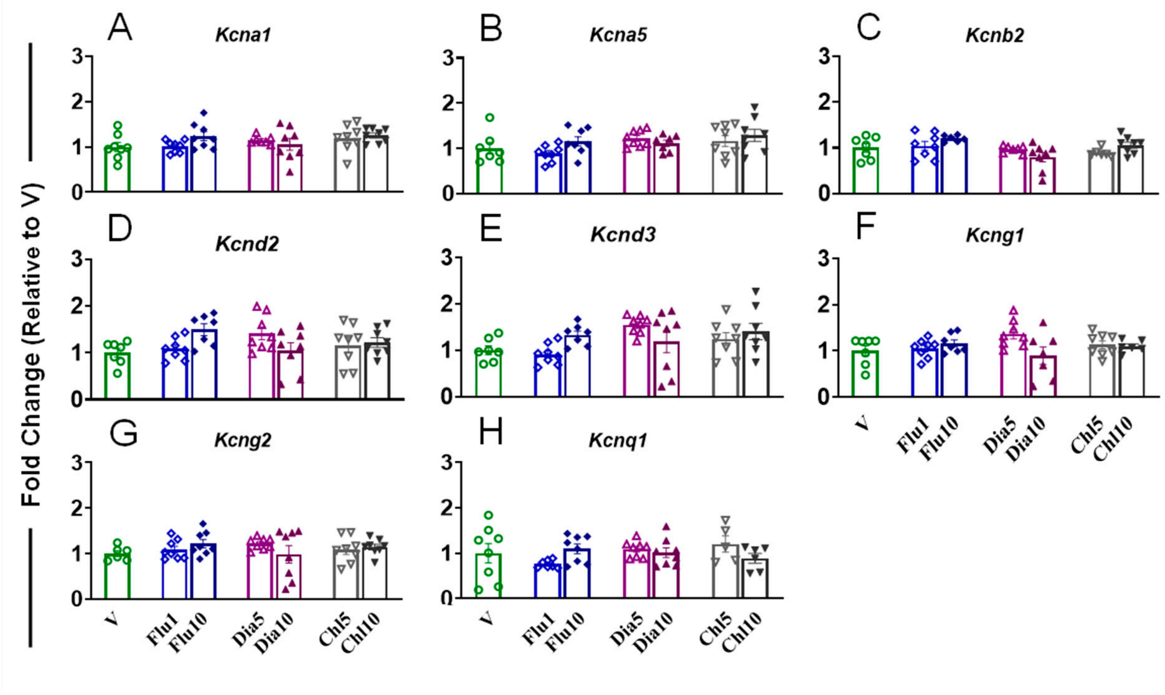

Figure S5. The effects of potassium agonist on the mRNA expression of voltage-gated potassium channels A) *Kcna1*; B) *Kcna5*; C) *Kcnb2*; D) *Kcnd2*; E) *Kcnd3*; F) *Kcng1*; G) *Kcn22*; and H) *Kcnq1* in the vHIP. Legend Key; vehicle control, V; flupirtine (1 mg/kg), Flu1; flupirtine (10 mg/kg), Flu10; diazoxide (5 mg/kg), Dia5; diazoxide (10 mg/kg), Dia10; chlorzoxazone (5 mg/kg), Chl5; chlorzoxazone (10 mg/kg), Chl10. Key to statistics -- no change.
